# Prediction of Real-World Long-Term Outcomes of People with CF Homozygous for the F508del Mutation Treated with CFTR Modulators

**DOI:** 10.3390/jpm11121376

**Published:** 2021-12-16

**Authors:** Danya Muilwijk, Marlou Bierlaagh, Peter van Mourik, Jasmijn Kraaijkamp, Renske van der Meer, Rutger van den Bor, Harry Heijerman, René Eijkemans, Jeffrey Beekman, Kors van der Ent

**Affiliations:** 1 Department of Pediatric Pulmonology, University Medical Center Utrecht, loc. Wilhelmina Children’s Hospital, Lundlaan 6, 3584 EA Utrecht, The Netherlands; d.muilwijk@umcutrecht.nl (D.M.); m.c.bierlaagh@umcutrecht.nl (M.B.); p.vanmourik-2@umcutrecht.nl (P.v.M.); j.kraaijkamp@students.uu.nl (J.K.); j.beekman@umcutrecht.nl (J.B.); 2 Department of Pulmonology, Haga Teaching Hospital, Els Borst-Eilersplein 275, 2545 CH The Hague, The Netherlands; r.vandermeer@hagaziekenhuis.nl; 3 Department of Data Science and Biostatistics, Julius Center for Health Sciences and Primary Care, University Medical Center Utrecht, 3584 EA Utrecht, The Netherlands; r.m.vandenbor@umcutrecht.nl (R.v.d.B.); m.j.c.eijkemans@umcutrecht.nl (R.E.); 4 Department of Pulmonology, University Medical Center Utrecht, Heidelberglaan 100, 3584 CX Utrecht, The Netherlands; h.g.m.heijerman@umcutrecht.nl; 5 Department of Regenerative Medicine, University Medical Center Utrecht, Uppsalalaan 8, 3584 CT Utrecht, The Netherlands

**Keywords:** Cystic Fibrosis, F508del/F508del, lumacaftor, tezacaftor, ivacaftor, forskolin-induced swelling, intestinal organoids, predictors, FEV1 decline, pulmonary exacerbations, sweat chloride concentration

## Abstract

The clinical response to cystic fibrosis transmembrane conductance regulator (CFTR) modulators is variable within people with cystic fibrosis (pwCF) homozygous for the F508del mutation. The prediction of clinical effect in individual patients would be useful to target therapy to those who would benefit from it. A multicenter observational cohort study was conducted including 97 pwCF (F508del/F508del), who started lumacaftor/ivacaftor (LUM/IVA) treatment before June 2018. In order to assess the associations of individual in vivo and in vitro biomarkers with clinical outcomes, we collected clinical data regarding sex, age, and sweat chloride concentration (SwCl) at baseline and after six months of LUM/IVA; the percent predicted forced expiratory volume in 1 s (ppFEV1) and the number of pulmonary exacerbations (PEx) during the three years before up to three years after modulator initiation; and the forskolin-induced swelling (FIS) responses to LUM/IVA, quantified in intestinal organoids. On a group level, the results showed an acute change in ppFEV1 after LUM/IVA initiation (2.34%, 95% CI 0.85–3.82, *p* = 0.003), but no significant change in annual ppFEV1 decline in the three years after LUM/IVA compared to the three years before (change: 0.11% per year, 95%CI: −1.94–2.19, *p* = 0.913). Neither of these two outcomes was associated with any of the candidate predictors on an individual level. The median number of pulmonary exacerbations (PEx) per patient year did not significantly change in the three years after LUM/IVA compared to the years before (median: 0.33/patient year, IQR: 0–0.67 before vs. median: 0/patient year, IQR: 0–0.67 after *p* = 0. 268). The PEx rate after modulator initiation was associated with the PEx rate before (IRR: 2.26, 95%CI: 1.67–3.08, *p* < 0.001), with sex (males vs. females IRR: 0.36, 95%CI: 0.21–0.63, *p* = 0.001) and with sweat chloride concentration (SwCl) at baseline (IRR: 0.96, 95%CI: 0.94–0.98, *p* = 0.001). The change in SwCl was also significant (−22.9 mmol/L (95%CI: −27.1–−18.8, *p* < 0.001) and was associated with SwCl at baseline (−0.64, 95%CI: −0.90–−0.37, *p* < 0.001) and with sex (males vs. females 8.32, 95%CI: 1.82–14.82, *p* = 0.013). In conclusion, ppFEV1 decline after CFTR modulator initiation remains difficult to predict in individual patients in a real-world setting, with limited effectiveness for double CFTR modulator therapies. The PEx rate prior to CFTR modulator treatment initiation, sex and SwCl at baseline could be potential predictors of long-term PEx rate and of changes in SwCl after modulator initiation.

## 1. Introduction

Cystic Fibrosis (CF) is the most prevalent autosomal recessive disorder, that affects over 90,000 people worldwide [1]. It is caused by mutations in the *cystic fibrosis transmembrane conductance regulator* (CFTR) gene, which encodes for an apically expressed anion channel in epithelial cells. The CFTR channel regulates fluid and electrolyte homeostasis of many mucosal surfaces [2]. The most common mutation is the deletion of the amino acid phenylalanine at position 508 (F508del), which is carried by approximately 85% of the global CF population. This mutation results in a misfolded CFTR protein with a strongly reduced apical trafficking and function. People with homozygous F508del mutations can benefit from small molecule combination therapy that targets the distinct defects of the F508del protein. The correctors lumacaftor (LUM, VX-809) and tezacaftor (TEZ, VX-661) enhance the processing and trafficking of the F508del-CFTR protein to the cell surface. Potentiators such as ivacaftor (IVA, VX-770) enhance the channel-opening probability (gating) and further increase the lumacaftor- or tezacaftor-corrected F508del function at the cell surface. Phase 3 clinical trials in people with CF (pwCF) homozygous for the F508del mutation demonstrated a modest efficacy of LUM/IVA or TEZ/IVA, as indicated by a 2.6–4% absolute increase in percent predicted forced expiratory volume in 1 s (ppFEV1) after 24 weeks of treatment, accompanied by a reduction in the pulmonary exacerbation (PEx) rate and sweat chloride concentration (SwCl) [3,4]. Sustained efficacy of treatment was demonstrated in phase 3 open-label extension trials with a two-year follow-up. These trials showed an average absolute reduction in ppFEV1 of 1–1.4% per year [5,6] and a 30–39% lower annualized PEx rate [3,4,5,6]. There is, however, a substantial variability in individual clinical response to CFTR modulators and the reason for this remains unknown. This variability has also been observed for newer and more effective triple therapy (elexacaftor/tezacaftor/ivacaftor) in people with F508del [7]. Predicting the CFTR modulator response in pwCF based on individual characteristics and in vivo or in vitro biomarkers would be useful in order to target costly therapies towards those patients who would benefit.

Biomarkers of CFTR function have been studied for their ability to predict individual clinical response to CFTR modulators. Several studies that focused on forskolin-induced swelling (FIS) of intestinal organoids found strong correlations between the average in vitro FIS response to CFTR modulators and short-term clinical drug response across groups with different genotypes [8,9] and in individuals with a variety of CFTR mutations [10]. These results raised interest in using the FIS assay as a biomarker to predict individual clinical responses to CFTR modulating therapies. Within pwCF homozygous for F508del, two small studies failed to predict the individual short-term clinical response to lumacaftor/ivacaftor (LUM/IVA) based on in vitro biomarkers such as FIS, nasal potential difference (NPD), intestinal current measurement (ICM) [11,12], β-adrenergic sweat secretion and serum drug concentration [12]. Other exploratory studies also did not detect an association between individual FIS response and short-term clinical response to LUM/IVA in pwCF carrying an A455E mutation [13] or to IVA in people with residual CFTR-function mutations [14]. Despite significant group-level responses in SwCl [11,12,13,14] or ppFEV1 [12,14], correlations between clinical endpoints were absent [12] or not reported [11,13,14].

In this real-world observational cohort study in pwCF homozygous for F508del, we assessed whether long-term ppFEV1 decline, PEx rate and SwCl change in response to LUM/IVA and whether long-term individual outcomes can be predicted by a combination of in vivo and in vitro biomarkers.

## 2. Materials and Methods

### 2.1. Study Design and Population

This multicenter observational cohort study was conducted in the CF centers of the University Medical Center Utrecht (UMCU) and Haga Teaching Hospital in The Hague, both in the Netherlands. PwCF homozygous for the F508del mutation were eligible for this study if CFTR modulating treatment with LUM/IVA had been initiated before July 2018 and individual intestinal organoids had been collected and stored in a biobank prior to CFTR modulator treatment. No exclusion criteria were specified. Total clinical follow-up was six years, starting from three years before up to three years after treatment initiation, or until censoring in case of (1) treatment discontinuation due to adverse events, (2) transition to elexacaftor/tezacaftor/ivacaftor (ELEX/TEZ/IVA), (3) lung transplantation, (4) death or (5) participants being lost to follow-up. Transition to TEZ/IVA during the study period was accepted, as its efficacy was considered to be comparable to LUM/IVA [3,4,5,6]. Written informed consent was obtained from all participants. The study was approved by the institutional review board (IRB) of the UMCU (IRB #16-668, TcBio #14-008).

### 2.2. Study Parameters

#### 2.2.1. Outcomes

The primary outcome was defined as change in average annual lung function decline in the first three years after LUM/IVA initiation, compared to the decline in the three years prior to LUM/IVA. Lung function was expressed as ppFEV1, calculated according to Global Lung Initiative (GLI) guidelines [15]; ppFEV1 was routinely measured every 3–6 months.

An acute change in ppFEV1 after LUM/IVA initiation, the total number of pulmonary exacerbations (PEx) requiring intravenous (IV) antibiotics during the first three years after LUM/IVA and a change from baseline SwCl (mmol/L) six months after LUM/IVA initiation were defined as secondary outcomes.

#### 2.2.2. Candidate Predictors

FIS response to LUM/IVA, defined as forskolin-induced organoid swelling after incubation with 3 µM LUM/IVA and 0.128 µM forskolin, quantified as area under the curve (AUC), was selected as a potential predictor of interest based on prior research. For all outcomes, the following candidate predictors were included based on previous literature and availability: total number of PEx requiring IV antibiotics during the three years before LUM/IVA treatment; sex (male/female); age at baseline, defined as age at date of treatment initiation; and SwCl at baseline (mmol/L), defined as the most recent SwCl value before LUM/IVA initiation. Average ppFEV1 decline during three years prior to LUM/IVA treatment was also included as a potential predictor of the primary outcome, whereas ppFEV1 at baseline was included for the secondary outcomes.

### 2.3. Study Procedures

#### 2.3.1. Clinical Data Collection

Data on clinical study parameters were retrieved from electronic medical records. We collected all available ppFEV1 measurements within the follow-up period. Total number of PEx was counted based on the start and stop dates of IV antibiotic courses in the three years before and three years after LUM/IVA initiation. Additional data were collected regarding type of CFTR modulating treatment and date and reason of censoring, if applicable. Date of treatment initiation was defined by the first start date of LUM/IVA. If LUM/IVA was discontinued within 3 months after the first initiation and/or for at least six months, date of treatment initiation was defined by the re-introduction date of LUM/IVA.

#### 2.3.2. Organoid Cultures and Measurements

All procedures regarding organoid culturing and measurements were performed by HUB Organoid Technology in the Netherlands.

The isolation of crypts out of rectal biopsies, the establishment of intestinal organoids and the FIS assays were performed according to previously described methods [8,16,17]. For the FIS assays, organoids were disrupted and seeded in a 96-well plate with an optical bottom (30–60 organoids per well). Immediately after seeding, 3 µM lumacaftor (VX-809) was added. After 24 h, organoids were stained with 10 µM calcein green, and 3 µM ivacaftor (VX-770) and forskolin (0.128 µM) were added. For each organoid model, technical duplicates and biological triplicates were performed (*n* = 6 datapoints). Organoid size was measured by fluorescence microscopy for a period of 60 min, taking images every 10 min with the Perkin Elmer Operetta CLS microscope. The resulting images were analyzed using Fiji (Fiji Life-Line version, 25 November 2014), an open-source image processing package based on ImageJ. HUB generated a script which recognizes organoids and quantifies change in size over time. The script identifies objects (organoids) and measures the area of each object at each time point. Subsequently, we calculated the change in size over time (relative to *t* = 0) for each object and the median change of size for each time point. The area under the curve (AUC) of the relative organoid size over time curves was calculated as the cumulative positive area between each two adjacent data points (size at *t* = 0).

### 2.4. Statistical Analysis

Baseline characteristics of the study population were summarized with descriptive statistics.

A multivariable linear mixed effects model was used to estimate ppFEV1 decline over time, ranging from −3 years to +3 years, with time = 0 (baseline) set at the date of LUM/IVA initiation. The model included a random intercept per subject and a random slope for time, CFTR modulator treatment, and the interaction between time and CFTR modulator treatment, assuming an unstructured covariance matrix. As fixed effects, we included time (in years) as a continuous variable; CFTR modulator, indicating CFTR modulator treatment status at the time of each ppFEV1 measurement (0 = no CFTR modulator, 1 = LUM/IVA or TEZ/IVA in case of transition during the study period); age at baseline; SwCl at baseline; sex; total number of PEx in the three years before CFTR modulator treatment; and FIS response to LUM/IVA. Moreover, an interaction term for time and CFTR modulator treatment (time: CFTR modulator) was added to the model, representing the change in ppFEV1 decline in the years after LUM/IVA. Subsequently, we used stepwise forward selection to test all other model covariates as a two-way interaction with time to assess whether ppFEV1 decline before CFTR modulator treatment was associated with covariate status, and as a two-way interaction with CFTR modulator to determine whether candidate predictors were associated with the acute change in ppFEV1 after LUM/IVA initiation. Finally, candidate predictors were tested as a three-way interaction with time: CFTR modulator, in order to identify predictors of change in ppFEV1 decline after LUM/IVA initiation. Model performance was assessed based on conditional and marginal R^2^.

Subsequently, we used a multivariable negative binomial model to identify predictors of the total number of PEx in the three years after LUM/IVA initiation. This model included total number of PEx in the three years after treatment as an outcome variable, with sex, age at baseline, SwCl at baseline, ppFEV1 at baseline, (log-transformed) number of PEx in the three years before LUM/IVA and FIS response to LUM/IVA as potential predictors. Total follow-up time (in years) after LUM/IVA initiation was used as offset. Model performance was assessed based on Nagelkerke’s R^2^.

Finally, the change in SwCl after LUM/IVA was analyzed with a multivariable linear regression model, including SwCl at baseline, age at baseline, ppFEV1 at baseline, sex, PEx in the three years before treatment initiation and FIS response to LUM/IVA as predictor variables. Adjusted R^2^ was used to describe model performance.

All analyses were performed in complete cases, given the low proportion of missing data (3% missing SwCl at baseline, 7% missing SwCl after LUM/IVA). *p*-values < 0.05 were considered statistically significant. Statistical packages lme4, lmerTest, MASS and Performance of R version 4.1.1 for Mac were used for the analyses.

## 3. Results

### 3.1. Study Population

In total, 97 pwCF with the F508del/F508del mutation were included in this study. Mean follow-up time was 3.2 years (±0.6 SD) before and 2.7 years (±0.7 SD) after LUM/IVA initiation. Censoring occurred in 12 participants due to transition to ELEX/TEZ/IVA (*n* = 9), treatment discontinuation (*n* = 1) or being lost to follow-up (*n* = 2). As summarized in Table 1, a substantial proportion (68%) of the study population transitioned to TEZ/IVA during the follow-up period, which was on average after two years of treatment with LUM/IVA (mean 1.9 years ± 0.5 SD). Over the entire study period, 2332 ppFEV1 measurements were collected from all participants. SwCl at baseline and SwCl after LUM/IVA were missing in 3 (3%) and 7 (7%) participants, respectively.

### 3.2. ppFEV1 and Change in ppFEV1 Decline

A multivariable linear mixed effects model was used to assess ppFEV1 decline over the entire observation period and to identify predictors of the acute change in ppFEV1 and of ppFEV1 decline in the three years after LUM/IVA initiation. Three participants were excluded from the analysis due to missing SwCl at baseline. As shown in Table 2, average annual ppFEV1 decline before LUM/IVA initiation was −2.14% per year (95% CI 3.77–−0.51, *p* = 0.012). A significant acute improvement of ppFEV1 was observed after LUM/IVA initiation (2.34%, 95% CI 0.85–3.82, *p* = 0.003), but the average annual ppFEV1 decline over three years did not change compared to the years before (change in decline: 0.11% per year, 95% CI −1.94–2.19, *p* = 0.913).

**Figure 1:**
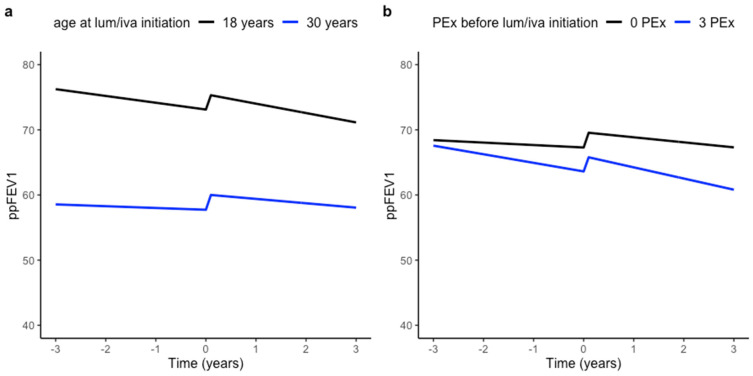
Predicted ppFEV1 decline before and after CFTR modulator initiation at varying ages and varying numbers of PEx. Plots are based on the linear mixed effects model coefficients in Table 2 to illustrate the associations of ppFEV1 decline with age at baseline (**a**) and with PEx (**b**). Time ranges from −3 years before to +3 years after LUM/IVA initiation, with time = 0 (baseline) defined by the start date of treatment with LUM/IVA. Model estimates suggested a faster ppFEV1 decline at a younger age which diminished at an older age. This is illustrated for an age at baseline of 18 years and 30 years (**a**), while all other covariates were kept constant at their mean or median values or at the reference category (as reported in Table 1). In addition, predicted ppFEV1 decline was plotted for pwCF without PEx vs. with 3 PEx in the three years before LUM/IVA initiation (**b**), to illustrate that predicted ppFEV1 decline may deteriorate with an increasing number of PEx. Model performance: conditional R^2^ 0.95, marginal R^2^ 0.34.

We determined whether candidate predictors (sex, age, SwCl, PEx and FIS response to LUM/IVA) were associated with average ppFEV1 and ppFEV1 decline before treatment with LUM/IVA. Age and SwCl at baseline demonstrated a significant association with average ppFEV1 (−1.28, 95% CI −1.66–−0.91, *p* < 0.001 and 0.32, 95% CI 0.06–0.58, *p* = 0.017, respectively). Age at baseline and total number of PEx were associated with ppFEV1 decline before LUM/IVA initiation and were therefore included in the multivariable linear mixed effects model (Table 2). Figure 1a illustrates that predicted ppFEV1 decline before LUM/IVA was on average 0.06% per year (95% CI 0.02–0.11, *p* = 0.004, Table 2) less for every additional year in age at baseline. In addition, predicted annual ppFEV1 decline before LUM/IVA was on average 0.31% per year (95% CI −0.48–−0.14, *p* < 0.001, Table 2) stronger per experienced PEx (Figure 1b). In contrast to the moderate significant acute improvement after LUM/IVA initiation, the three-year average annual ppFEV1 decline did not change on a group level when comparing trends before and after LUM/IVA treatment. Nevertheless, we did not find an association of either the acute change in ppFEV1 or annual ppFEV1 decline in the three years after LUM/IVA initiation with the candidate predictors, which were left out of the model in order to reduce the complexity of the model and to improve the performance (conditional R^2^ 0.95, marginal R^2^ 0.34).

### 3.3. Pulmonary Exacerbations

Overall, the median number of PEx requiring IV antibiotics in the three years before LUM/IVA initiation was 0.33 (IQR: 0–0.67) per patient year, which did not significantly change in the three years after (median: 0, IQR: 0–0.67, *p* = 0.268). Predictors of the absolute number of PEx during the first three years of treatment with LUM/IVA were assessed with a negative binomial model. Three participants were excluded due to missing SwCl at baseline.

**Figure 2:**
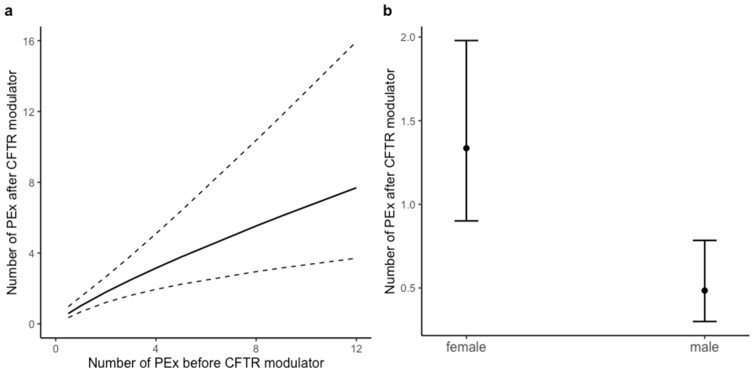
Association of PEx in the three years after LUM/IVA initiation with the number of PEx three years before LUM/IVA (**a**) and the difference in PEx after LUM/IVA between females and males (**b**). Predicted associations in this figure are illustrated on the original scale, based on the incidence rate ratios (IRR) in Table 3. All other covariates were kept constant at their mean or median values or at the reference category (as reported in Table 1). Dashed lines in Figure 2a and error bars in (**b**) represent 95% confidence intervals. Model performance: Nagelkerke’s R^2^ = 0.60.

The number of PEx after LUM/IVA was associated with the (log-transformed) number of PEx before LUM/IVA, with sex and SwCl at baseline (Table 3). The predicted relationship between the number of PEx before and after LUM/IVA on the original scale is illustrated in Figure 2a. Relative rate of PEx in males was three times lower (IRR 0.36, 95% CI 0.21–0.63, *p* < 0.001) compared to females (Figure 2b). SwCl at baseline was also significantly associated with the number of PEx after LUM/IVA (IRR 0.96, 95% CI 0.94–0.98, *p* = 0.001), but not with age at baseline, ppFEV1 at baseline or FIS response to LUM/IVA.

### 3.4. Change in Sweat Chloride Concentration

**Figure 3:**
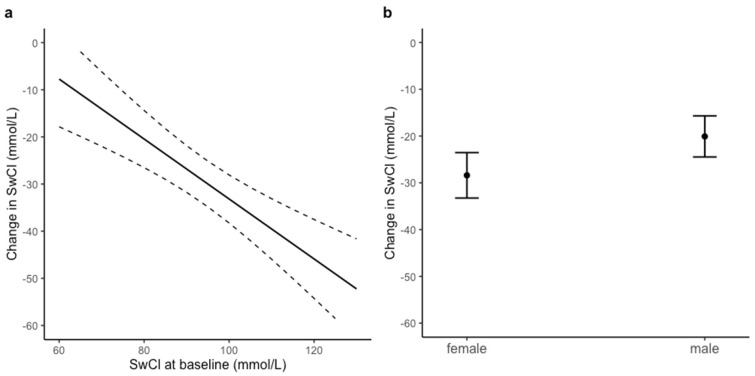
Association of change in SwCl six months after LUM/IVA with SwCl at baseline (**a**) and the difference in SwCl change between females and males (**b**). Predicted associations in this figure are illustrated according to model coefficients in Table 4. All other covariates were kept constant at their mean or median values or at the reference category (as reported in Table 1). Dashed lines in (**a**) and error bars in (**b**) represent 95% confidence intervals. Model performance: adjusted R^2^ = 0.26.

SwCl levels significantly improved after approximately six months (mean 7.2 months ± 4.8 SD) of treatment with LUM/IVA, with an average absolute change from baseline of −22.9 mmol/L (95% CI −27.1–−18.8, *p* < 0.001). Candidate predictors of change in SwCl were assessed by means of linear regression. We excluded 9 participants due to missing SwCl at baseline or SwCl after treatment initiation. Table 4 shows that the change in SwCl after LUM/IVA was associated with SwCl at baseline (−0.64, 95% CI −0.90–−0.37, *p* < 0.001) and sex (8.32, 95% CI 1.82–14.82, *p* = 0.013). As illustrated in Figure 3, this suggested that the decrease in SwCl was greater in participants with higher baseline SwCl levels (Figure 3a) and smaller in males compared to females (Figure 3b). The change in SwCl after treatment initiation was not associated with baseline ppFEV1, PEx or FIS response to LUM/IVA.

## 4. Discussion

The aim of the study presented here was to assess the real-world long-term clinical effectiveness of double CFTR modulator therapies in pwCF homozygous for the F508del mutation and to assess the association of several in vivo and in vitro parameters with clinical endpoints, in order to determine whether these parameters could serve as predictors of long-term treatment response to CFTR modulators in a real-world setting.

This study did not show a significant improvement in ppFEV1 decline or the number of PEx, when comparing three years before and after treatment with LUM/IVA. However, the data did indicate an overall acute ppFEV1 improvement and a decline in SwCl concentration after six months, consistent with previous observations in clinical trials [3,4,5,6]. Our results of ppFEV1 decline were different from the original phase 3 open-label extension study of LUM/IVA in pwCF homozygous for the F508del mutation, which showed an average annual ppFEV1 decline of −1.3% compared to −2.3% in untreated matched historical controls after 120 weeks of treatment [5]. This is probably related to our real-world approach, which had no restrictions regarding age or baseline ppFEV1 and a longer follow-up period before and after treatment initiation. One other study reported a moderate improvement of ppFEV1 decline after one year of LUM/IVA in a real-world setting [18], which is in line with the short-term average improvement of 2.3% found in our study population and in the first short-term clinical trials [3,4]. These findings underline the risk of extrapolating data from controlled studies into daily clinical practice. Alternatively, the real-world setting may be responsible for stronger variations in ppFEV1 measurements. We aimed to reduce the impact of measurement variability of ppFEV1 by including multiple repeated measurements, but other unmeasured sources of variation in ppFEV1 may play an important role [19].

Despite the absence of an overall group-level change in ppFEV1 decline, associations at the individual level could still be demonstrated when substantial individual variation is present. This study showed that ppFEV1 decline before CFTR modulator use was associated with age and number of PEx, but we could demonstrate neither an association between any of the studied parameters and the acute change in ppFEV1, nor an association between the parameters and the change in long-term ppFEV1 decline after CFTR modulator initiation. This suggests that the individual variation in both the acute change in ppFEV1 and the change in ppFEV1 decline might have been too low in combination with the limited effectiveness of LUM/IVA.

The number of PEx in the three years before CFTR modulator treatment and sex were associated with the number of PEx in the three years after CFTR modulator initiation. This is in accordance with several studies reporting worse pulmonary outcomes and a higher mortality risk in females [20,21,22], despite equal levels of care between males and females [23]. The so-called ‘gender-gap’ in pwCF has already been described for many years and the cause is probably multifactorial. The level of female sex hormones may play an important role in the severity of CF lung disease, as it influences mucociliary clearance, infection and inflammation, which ultimately leads to a higher frequency of PEx and a more rapid deterioration of lung function [24]. Since our results did not show a significant reduction in the number of PEx after LUM/IVA initiation compared to the years before treatment, this could also reflect prognostic differences related to disease severity, which may indicate that those with severe disease manifestations might also remain the most affected patients after treatment initiation.

We found a different association between sex and change in SwCl after six months of LUM/IVA, with a greater reduction in SwCl in females compared to males. So far, few studies have focused on the differential effect of CFTR modulators between sexes on clinical outcomes. One study in a small group of pwCF with severe lung disease (ppFEV1 < 40%) described no differences in PEx rate between males and females one year after commencement with LUM/IVA [25]. However, a greater reduction in both SwCl and PEx rate was observed in females carrying CFTR-gating mutations after two years of treatment with IVA [26]. These contradictory results emphasize that additional research is warranted to further elucidate the effects of suggested sex differences on long-term outcomes. Finally, the negative association between SwCl at baseline and the number of PEx in the years after LUM/IVA initiation, although it was very weak, was opposed to existing literature, which has reported that higher levels of SwCl correspond to a more severe CF phenotype [27,28]. The decreasing number of exacerbations after LUM/IVA with increasing baseline SwCl levels are, therefore, difficult to explain and might have been influenced by measurement variation in SwCl [29].

To date, only a few studies have focused on the association of in vitro biomarkers such as FIS, ICM and NPD with short-term changes in ppFEV1 and SwCl as parameters of clinical response to CFTR modulators. Even though residual CFTR function measured by FIS was correlated with disease severity of pwCF homozygous for F508del [28], our results were in line with other exploratory studies, which were also not able to detect associations between individual short-term clinical response and in vitro biomarkers quantifying response to CFTR modulators [13,14,15]. This is likely explained by the limited effectiveness of LUM/IVA and the relatively low individual variation in the measured clinical outcomes in a real-world setting in a homogeneous group of F508del/F508del pwCF. Other studies, which demonstrated a strong association of FIS with short-term changes in SwCl and ppFEV1 on a group level and on the individual level, included pwCF with a variety of CFTR mutations and a wider range of clinical responses [8,9,10]. Future research might therefore focus on the prediction of clinical response to highly effective CFTR modulators such as ELEX/TEZ/IVA or other more potent therapies, and may include people with a variety of CFTR mutations to identify clinical responders and facilitate personalized treatment.

The retrospective observational design of this real-world before-after study could be regarded as a limitation of this study, although all study parameters were systematically collected as part of standard clinical care with a low proportion of missing data. In addition, a substantial proportion (63%) of our participants transitioned to TEZ/IVA after approximately two years of treatment with LUM/IVA. This could have influenced the results, but we expect that this did not over- or underestimate the change in ppFEV1 decline given the comparable efficacy of these CFTR modulators [4,6]. Unfortunately, the number of selected candidate predictors was restricted by the sample size. Larger prospective studies including more candidate predictors would be required to be able to develop and validate a clinical prediction model.

In summary, our study showed a limited overall effectiveness of double CFTR modulator therapy in pwCF homozygous for F508del after three years. Individual prediction of long-term clinical response remains difficult in a real-world setting, although PEx rate prior to CFTR modulator treatment initiation, sex and SwCl at baseline could be potential predictors of long-term PEx rate and of changes in SwCl after modulator initiation.

## Figures and Tables

**Table 1:** Baseline characteristics of the study population (*n* = 97).

CF center, *n* (%) University Medical Center Utrecht Haga Teaching Hospital The Hague	88 (91) 9 (9)
CFTR modulator, *n* (%) Lumacaftor/ivacaftor Tezacaftor/ivacaftor transition during follow-up	97 (100) 66 (68)
CFTR modulator treatment duration (years), mean (SD)	2.7 (0.7)
ppFEV1 three years before modulator (%), mean (SD)	69.6 (21.8)
ppFEV1 at baseline (%), mean (SD)	66.4 (22.0)
Number of PEx per patient year before modulator, median (IQR)	0.33 (0–0.67)
SwCl at baseline (mmol/L), mean (SD)	92.0 (13.1)
Female sex, *n* (%)	44 (45)
Age at baseline (years), median (IQR)	23.5 (17.0–31.1)
FIS response to LUM/IVA (AUC), median (IQR)	948.9 (647.9–1418.1)

CFTR: cystic fibrosis transmembrane conductance regulator protein. Baseline: defined as date of CFTR modulator initiation. ppFEV1: percent predicted forced expiratory volume in 1 s. Number of PEx: average number of pulmonary exacerbations (PEx) requiring intravenous (IV) antibiotics per patient year, in the three years before CFTR modulator initiation. SwCl: sweat chloride concentration. FIS response to LUM/IVA: corrected forskolin-induced swelling response of intestinal organoids to 3 µM lumacaftor/ivacaftor (LUM/IVA) and 0.128 µM forskolin minus the response to 0.128 µM forskolin alone, quantified as area under the curve (AUC) of the normalized organoid swelling over 1 h.

**Table 2:** Multivariable linear mixed effects model of ppFEV1 decline (*n* = 94, obs = 2233).

	Coefficient	95% CI	*p*-Value
Time	−2.14	−3.77–−0.51	0.012 *
CFTR modulator	2.34	0.85–3.82	0.003 *
Male sex	6.38	−0.29–13.06	0.069
Age at baseline	−1.28	−1.66–−0.91	<0.001 *
SwCl at baseline	0.32	0.06–0.58	0.017 *
Number of PEx	−1.22	−2.83–0.38	0.145
FIS response to LUM/IVA	0.19	−0.42–0.79	0.554
Time: age at baseline	0.06	0.02–0.11	0.004 *
Time: number of PEx	−0.31	−0.48–−0.14	<0.001 *
Time: CFTR modulator	0.11	−1.94–2.19	0.913

Definitions and abbreviations: Time is in years. CFTR modulator indicates treatment with LUM/IVA or TEZ/IVA (in case of transition during the study follow-up). Male sex is compared to the reference category female sex. Age is in years. SwCl is in mmol/L. Number of PEx: total number of PEx requiring IV antibiotics in the three years before CFTR modulator initiation. FIS response to LUM/IVA: corrected forskolin-induced swelling response of intestinal organoids to 3 µM lumacaftor/ivacaftor (LUM/IVA) and 0.128 µM forskolin minus the response to 0.128 µM forskolin alone, quantified as area under the curve (AUC) of the normalized organoid swelling over 1 h, scaled 1:100. Model performance: conditional R^2^ 0.95, marginal R^2^ 0.34. * Significance level *p* < 0.05. Interpretation: The coefficient CFTR modulator represents the acute change in average ppFEV1 directly after modulator initiation. The coefficients of male sex, age at baseline, SwCl at baseline, number of PEx and FIS response to LUM/IVA illustrate the associations with average ppFEV1. Coefficients of time:age at baseline and time:number of PEx define the association of age and PEx with ppFEV1 decline before CFTR modulator initiation. Time:CFTR modulator indicates the average change in ppFEV1 decline after CFTR modulator initiation compared to the ppFEV1 decline before modulator use.

**Table 3:** Multivariable negative binomial model of total number of PEx requiring IV antibiotics in the first three years after LUM/IVA initiation (*n* = 94).

	Coefficient	IRR	95% CI (IRR)	*p*-Value
Log (number of PEx)	0.81	2.26	1.67–3.08	<0.001 *
Male sex	−1.01	0.36	0.21–0.63	<0.001 *
Age at baseline	0.03	1.03	0.99–1.06	0.125
SwCl at baseline	−0.04	0.96	0.94–0.98	0.001 *
ppFEV1 at baseline	−0.01	0.99	0.98–1.01	0.467
FIS response to LUM/IVA	0.01	1.01	0.69–1.06	0.706

Coefficients are on the log-scale. The incidence rate ratios (IRR) are the coefficients transformed back to the original scale and represent the relative change in the number of PEx in the three years after CFTR modulator initiation for every 1-unit change of the continuous variables, or for male sex compared to the reference category female sex. Model performance: Nagelkerke’s R^2^ = 0.60. * Significance level *p* < 0.05.

**Table 4:** Multivariable linear regression model of absolute change in SwCl after LUM/IVA (*n* = 88).

	Coefficient	95% CI	*p*-Value
SwCl at baseline	−0.64	−0.90–−0.37	<0.001 *
Age at baseline	0.05	−0.37–0.47	0.822
ppFEV1 at baseline	−0.02	−0.20–0.15	0.786
Male sex	8.32	1.82–14.82	0.013 *
Number of PEx	−0.40	−1.95–1.15	0.612
FIS response to LUM/IVA	0.22	−0.31–0.74	0.411

Model coefficients represent the predicted change in SwCl for every 1-unit change of the continuous variables or for male sex compared to female sex (which is the reference category). Model performance: adjusted R^2^: 0.26. * Significance level *p* < 0.05.

## Data Availability

The data presented in this study are available on request from the corresponding author, in consultation with HUB Organoid Technology.
